# A Treatise on Mental Diseases

**Published:** 1901-01

**Authors:** 


A Treatise on Mental Diseases. Based upon the lecture course at the Johns Hopkins University, 1899, and designed for the use of practitioners and students of medicine. By Henry J. Berkley, M. D., Clinical Professor of Psychiatry, Johns Hopkins University, etc Octavo, pp. xiv.—601. Illustrated. New York: D. Appleton & Co. 1900.

The addition of a new work on mental diseases would ordinarily attract little attention from the reviewer. In no branch of medical literature do we find greater sameness in description and method of subjects. Of late we have had many excellent works upon insanity. The plan of these has been mainly that of careful classification according to clinical types. The description of types of insanity has left nothing to be desired. In this volume we notice with pleasure a distinct departure in method. We see the direct effect of the close pathological study that has been done in the last ten years.

In the section on special pathology, Berkley presents, as it has not before been presented in an American clinical treatise on insanity, the special pathology of the nerve cell. He reviews the results obtained in the study of the changes in the granula and achromatic substance of the cell by the methyline blue method. The manner of experimentation by section of the root fibres and by poison effects is clearly described and the results of a host of experimenters are presented. Of the chrome silver method of staining the entire neurone as developed by Golgi, he says that this has not been prolific of good results when applied to the cortex of man. By this method the cellular changes shown are limited to lesions of a gross character, none of the finer detail of the nucleus or protoplasm being brought into view.

The pathology of cerebral bloodvessels is clearly presented and its value increased by several good illustrations of arterial changes in different specimens reproduced by photographs.

In the classification of mental diseases he has followed Krafft-Ebing and divides insanity into four groups.

Group 1, mental diseases without ascertainable pathological alteration of the brain substance. This includes acquired forms of insanity arising in individuals without an inherited tendancy to insanity. The chief causes are physical diseases and shock. These are the cases most benefited by therapeutic measures.

Group 2—mental diseases sequential to ascertainable alterations of the cerebral substance. In this group the mental symptoms are the result of lesion of the neural tissues. They are secondary to organic degenerative or inflammatory processes beginning in the bloodvessels of the cerebral pulp.

Group 3—insanities due to inherited or acquired mental instability. These are the cases in whom we find defective development of the cranial bones, imperfections in the brain convolutions. This class is very large and probably includes more cases than any of the other groups.

Group 4—states of complete or incomplete retardation of the psychical and physical development. These cases depend upon gross deficiences in the development of the brain and also in the lack of some essential glandular secretion, or upon a cerebral hemorrhage, trauma or infectious processes occurring in the earliest years of life, which have prevented the perfect evolution of the faculties by retarding or stopping brain growth.

In the treatment of insanities the author is firm in his belief that the milder cases of the acute and chronic forms should be treated at home. The description of the general well-known forms of insanity are graphic and will give the reader as good an idea of insanity as can be obtained from any book.

Altogether we are very much impressed by the general excellence of this book. The paper, type and illustrations are all of a quality that add much to its value. As a book of reference it is fully up to date and will meet the requirements of the general practitioner and specialist. J. W. P.

